# Chiral Nanostructured Glycerohydrogel Sol–Gel Plates of Chitosan L- and D-Aspartate: Supramolecular Ordering and Optical Properties

**DOI:** 10.3390/gels10070427

**Published:** 2024-06-28

**Authors:** Anna B. Shipovskaya, Olga S. Ushakova, Sergei S. Volchkov, Xenia M. Shipenok, Sergei L. Shmakov, Natalia O. Gegel, Andrey M. Burov

**Affiliations:** 1 Institute of Chemistry, Saratov State University, Saratov 410012, Russia; shipovskayaab@yandex.ru (A.B.S.); olgakol4ina777@yandex.ru (O.S.U.); kshipenok@gmail.com (X.M.S.); gegelno@yandex.ru (N.O.G.); 2 Department of Physics, Yuri Gagarin Saratov State Technical University, Saratov 410054, Russia; volchkov93@bk.ru; 3 Saratov Branch, Institute of Radio Engineering and Electronics of Russian Academy of Sciences, 38 Zelyonaya St., Saratov 410019, Russia; 4 Institute of Biochemistry and Physiology of Plants and Microorganisms, Saratov Scientific Centre of the Russian Academy of Sciences, Saratov 410049, Russia; burov.anmi@gmail.com

**Keywords:** chitosan, glycerohydrogels, sol-gel synthesis, chiroptical properties, collimated transmission, X-ray diffraction, small-angle X-ray scattering, microscopy

## Abstract

A comprehensive study was performed on the supramolecular ordering and optical properties of thin nanostructured glycerohydrogel sol-gel plates based on chitosan L- and D-aspartate and their individual components in the X-ray, UV, visible, and IR ranges. Our comparative analysis of chiroptical characteristics, optical collimated transmittance, the average cosine of the scattering angle, microrelief and surface asymmetry, and the level of structuring shows a significant influence of the wavelength range of electromagnetic radiation and the enantiomeric form of aspartic acid on the functional characteristics of the sol-gel materials. At the macrolevel of the supramolecular organization, a complex topography of the surface layer and a dense amorphous–crystalline ordering of polymeric substances were revealed, while at the nanolevel, there were two forms of voluminous scattering domains: nanospheres with diameters of 60–120 nm (L-) and 45–55 nm (D-), anisometric particles of lengths within ~100–160 (L-) and ~85–125 nm (D-), and widths within ~10–20 (L-) and ~20–30 nm (D-). The effect of optical clearing on glass coated with a thin layer of chitosan L-(D-)aspartate in the near-UV region was discovered (observed for the first time for chitosan-based materials). The resulting nanocomposite shape-stable glycerohydrogels seem promising for sensorics and photonics.

## 1. Introduction

In recent years, the increasing scientific and practical interest of researchers has been aimed at creating thin-film nanostructured polymer composites, including gel films, for solving problems of materials science in new areas such as biodegradable electronics, sensorics, and photonics, including disposable production products. The aminopolysaccharide chitosan (CS), belonging to the class of biodegradable chiral high-molecular-weight substances, is considered a promising polymer for obtaining an optically sensitive layer of hydrogel coatings with nanosized supramolecular ordering. It easily dissolves in a slightly acid water medium and is obtained from annually renewable natural raw materials, which contributes to the development of environmentally friendly technologies to minimize negative effects on the environment. The possible uses of such promising materials are listed in Table 1.

To obtain nanostructured sensitive layers of optical materials from CS, inorganic nanoparticles are introduced into the polymer matrix. For example, biodegradable CS nanocomposites with graphene oxide (GO) exhibit a semiconductor nature and ohmic-type conductivity [18]. The optical band gap and ohmic contacts are governed by varying the content of GO nanoparticles in the material. The formation of GO nanocomposites based on double spatial networks of CS with polyacrylamide provides synergistic improvement of the mechanical properties of the multihydrogel structure [19]. This enhancement is explained by increased energy dissipation due to the more intense unfolding of conjugated polymer chains during deformation. By incorporating copper nanowires into CS via simple sol–gel transition, biodegradable, ultra-flexible, transparent, and conductive films with good optical and electrical properties are obtained [4]. The efficiency of such nanocomposites exceeds that of transparent thermoplastic synthetic polymers filled with Cu or Ag nanowires, carbon nanotubes, or graphene [20,21,22]. When copper oxide (CuO) nanoparticles are introduced into a polymeric film made of a CS and polyethylene oxide mixture, the dielectric constant, dielectric losses, and alternating current conductivity decrease [23]. Moreover, the introduction of CuO nanoparticles leads to an increase in the crystallinity and roughness parameters (proportional to the numbers of active centers in the polymeric mixture) of nanocomposites, as well as a bathochromic shift in the absorption maximum and a hypochromic effect in the optical density of the samples. It is also noted that the properties of sensors for detecting metal ions and biological structures based on the resonant excitation of the vibrations of surface free electrons are largely determined by the effective refractive index of CS [24]. Hydrogel films based on cross-linked CS could exhibit nonlinear optical properties, which opens up new directions for the practical application of chitosan-containing materials [25].

Recent research has shown that nanostructured hydrogel composites based on interpenetrating polymer networks of CS, the product of the hydrolysis and condensation of tetraethylorthosilicate (TEOS, a sol-gel precursor) and ordered colloidal silica, exhibit pH-dependent expansion of the SiO_2_ photonic crystal lattice, which is visually manifested in a change in the color of the composite upon swelling in media with different pH levels [26]. Structural-chiral plasmonic gold nanoparticles are very promising for designing photonic devices on flexible polymer substrates [8,27]. Based on these, a new type of random laser was proposed [27], whose radiation has a significantly high level of asymmetry between light with right and left circular polarization. This offers significant advantages in potential applications such as 3D optical metamaterials with controlled properties, particularly nano-photonic sensors, microdisplays, and holographic devices. Considering that CS belongs to the class of chiral D-glucans and the structural and conformational lability of its macrochains and supramolecular structures built therefrom, it seems that the use of the nanoparticles of this aminopolysaccharide could not only expand the range of optical applications of polymeric materials but also obtain chiral nanocomposites with novel functional properties.

Regarding CS chirality, we would note the following. In solution, CS macromolecules exhibit molecular conformational chirality due to asymmetrically substituted carbon atoms (chiral centers) in the elementary units and the unfolded (including helical) conformations of macrochains. In the solid-phase state, they also exhibit structural chirality, whose quantitative indicators are determined by the chemical form of the polymer (salt or base) [28,29,30]. Solutions of CS salts with achiral acids (HCl, CH_3_COOH, Na-acetate buffer), traditionally used to dissolve the polymer, exhibit no optically active electronic transitions either in the UV or visible range of the spectrum and are characterized by left-hand rotation of plane-polarized light [30]. Solutions of CS salts with chiral acid solvents or CS derivatives with chromophoric groups exhibit both left-hand and right-hand rotation, as well as a pronounced Cotton effect [29,31,32,33,34]. The chiro-optical properties of CS are used to design photonic nanostructured materials with tunable chiro-optical parameters for optical sensing and stenciling [35,36], e.g., hybrid organic–inorganic hydrogels based on CS salts with protonated amino groups ${\text{–}\mathrm{NH}}_{3}^{+}$), which lead to the high swelling degree of the material, and the TEOS polysiloxane network is formed as a result of the sol–gel synthesis of TEOS exhibiting pH-sensitive photonic properties, expressed as a shift in the diffracted wavelength and generating a pH-dependent color change [26]. Diffraction in the visible range depending on the pH of the medium was demonstrated by mixed hydrogels of achiral functionalized polyvinyl alcohol and colloidal CS [37].

In this work, CS solutions in L- and D-aspartic acid (L- and D-AspA) were used to prepare chiral nanostructured hydrogels. Its choice was due to the following reasons. Ionic complexation between CS and AspA occurs through the protonation reaction of –NH_2_ groups to form the aspartate complex of CS (CS·AspA) [29,38,39]. In the CS + AspA + H_2_O system, the effect of counterionic condensation with phase segregation of the salt form of the polymeric substance at a nanoparticle level was discovered [39,40], which predetermines the possibility of the one-stage formation of a chitosan-containing hydrogel with nanosized CS·AspA aggregates included therein. One AspA molecule contains two acidic –COOH groups and one basic –NH_2_ group. Therefore, in an aqueous medium, cationic, zwitterionic, anionic, and dianionic forms [41] exist in varying proportions depending on pH, the ratio between which could affect the protonation degree of CS (the average number of ${–\mathrm{NH}}_{3}^{+}$ groups per monomeric unit) and, accordingly, the morpho-dimensional characteristics of nanostructures. Both chiral enantiomers of L- and D-AspA are commercially available.

Note also that the crystallinity degree of CS·L-AspA, estimated by Lugovitskaya et al. [39], is significantly higher than that of the salt complexes of CS with classical monobasic inorganic and organic acids [42,43], which is apparently due to the formation of anhydrous crystalline modifications of the aminopolysaccharide [44,45]. The latter, in turn, should help to increase the level of nanostructuring in the polymeric system when it is fixed in a hydrogel material. Most likely, the compacted supramolecular structure of salts and ionic association processes should also be observed when using the D-enantiomer of the acid. It is important to discover this, since homochiral D-glucan·D-acid salts are characterized by a more energetically favorable chain conformation and, accordingly, a more equilibrium spatial structure than heterochiral D-glucan·L-acid salts (established by the example of diastereomeric CS salts with L- and D-ascorbic acid [46]).

To make a gel structure in the form of shape-stable thin-film plates, we used the approach tested for CS L- and D-ascorbates [47,48]. It consists of the formation of a system of interpenetrating spatial networks of organic and inorganic nature in one polymeric sample. The organic network is represented by a physical gel of CS and an inert structure former, whose –OH groups act as a template for the synthesis of an inorganic chemical network of silicon polyolate (~–Si–O–Si–~). Since, in our case, the glycerol solution of silicon tetraglycerol (Si(OGly)_4_ ∙ GlyOH) is the inorganic phase precursor, the sol–gel synthesis is accompanied by the formation of polymeric glycerohydrogels.

The purpose of this work was to study the supramolecular ordering and optical properties of chiral nanostructured glycerohydrogel sol-gel plates of chitosan L- and D-aspartate and their individual components in a wide spectral range of electromagnetic radiation to expand the area of their possible usage, including green technologies for sensorics and photonics.

## 2. Results and Discussion

**Figure 1:**
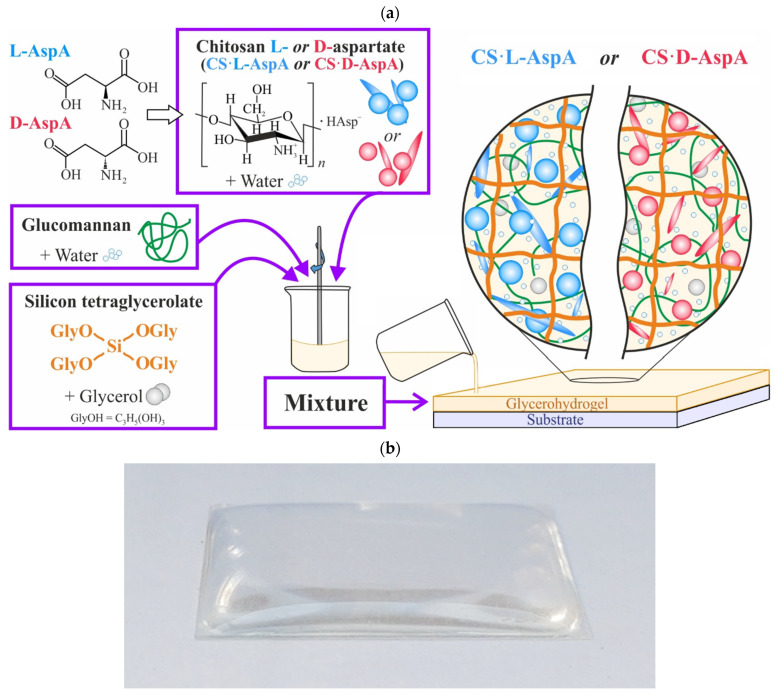
Scheme of the preparation process (**a**) and a photo (**b**) of our glycerohydrogel sol-gel plates based on CS·L-AspA and CS·D-AspA.

The process of obtaining glycerohydrogel sol-gel plates consisted of mixing solutions of CS·L-(D-)AspA, glucomannan, and Si(OGly)_4_ ∙ GlyOH, with subsequent gelation on a glass substrate (Figure 1a). In this case, hydrolysis of the Si–O–C bonds in Si(OGly)_4_ occurs in the mixed composition, accompanied by the sol-gel synthesis of a spatial network of Si polyolate [48], while glucomannan acts as a template for the condensation of ≡Si–OH groups into disiloxane groups ≡Si–O–Si≡.

The obtained samples of CS·L-(D-)AspA-based glycerohydrogels were shape-stable and relatively elastic thin-film plates (Figure 1b). Their optical properties and supramolecular ordering, as well as those of the individual polymer components included therein (solutions, thin films, and powders), were studied. Special attention was paid to assessing the influence of the isomeric form of the acid residue (L-(D-)HAsp^−^, Figure 1a) of the salt complexes on the characteristics of the samples.

### 2.1. Chiro-Optical Properties

In the first stage, the optical activity of L- and D-enantiomeric salts of CS aspartate was assessed by varying the molar ratio [AspA]:[–NH_2_] and in comparison with optically pure L- and D-antipodes of AspA. To control them, the specific optical activity and the light beam ellipticity of the samples were measured.

**Figure 2:**
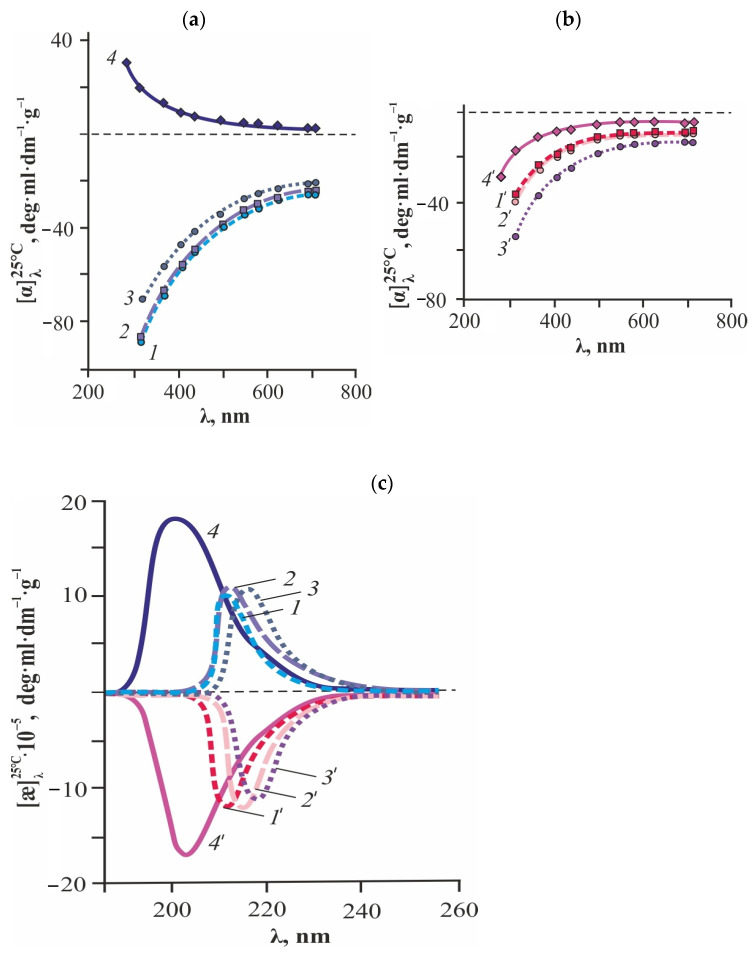
ORD curves (**a**,**b**) and CD spectra (**c**) of aqueous solutions of CS in L- (*1*–*3*) and D-AspA (*1*′–*3*′) at the molar ratio [AspA]:[–NH_2_] = 0.85 (*1*, *1*′), 1.0 (*2*, *2*′) and 2.0 mol·(mol of NH_2_)^−1^ (*3*, *3*′), as well as those of individual L-AspA (*4*) and D-AspA (*4*′).

Studies in the visible range of the spectrum have shown that the optical rotation dispersion (ORD) curves of aqueous solutions of CS·L-AspA and CS·D-AspA are smooth, characterized by left-hand rotation of the polarization plane, and belong to the normal type: negative [α] values monotonically decrease in magnitude with increasing λ (Figure 2a,b). The dispersion [α] = *f*(λ) of solutions of individual AspA enantiomers also reveals no optically active electronic transitions; however, L-AspA and D-AspA show right-hand and left-hand rotation, respectively. The observed nature of ORD for CS·L-(D-)AspA is due, on the one hand, to the predominant contribution to the total optical activity of the polymeric system from the molecular and structural conformational chirality of the salt CS form compared to the molecular enantiomeric chirality of the individual antipodes of the acid. On the other hand, it confirms the formation of a spatially close complex between the protonated amino groups of CS and the resonance-stabilized aspartate anion. With an increase in the molar ratio [AspA]:[–NH_2_], accompanied by a significant pH decrease (due to changes in the proportions of the cationic, zwitterionic, and cationic forms of aspartate ions) and a decrease in the protonation degree of the amino groups of the polymer (Table 2), the absolute value of [α] of CS∙L-AspA and CS∙D-AspA solutions decreases and increases, respectively. In all cases, the absolute values −[α] were significantly larger for the CS·L-AspA salt. After normalization to the acid concentration, the ORD curves of CS·L-(D-)AspA solutions with the ratio [AspA]:[–NH_2_] > 1 mol/mol of NH_2_ almost coincide with the spectra of the equimolar systems.

The curves of the circular dichroism (CD) of aqueous solutions of CS·L-AspA and CS·D-AspA exhibit a positive and negative Cotton effect, respectively, in the UV range (Figure 2c). This is the result of the excitation of the C=C bond in the aminoaspartate chromophore and corresponds to a π→π* transition. The interaction of the $–{\mathrm{NH}}_{3}^{+}$ groups of the aminopolysaccharide chain with HAsp^−^ is also evidenced by a decrease in the dichroic absorption intensity of CS∙L-(D-)AspA solutions compared to L-(D-)AspA. Note that this effect is not observed in aqueous solutions of low-molecular-weight CS and in solutions of CS in HCl, CH_3_COOH, or Na-acetate buffer, i.e., for CS salts with an achiral acid residue [32]. Like plane-polarized light in the visible range, for circularly polarized light in the UV range, the influence of the [AspA]:[–NH_2_] molar ratio and, accordingly, the protonation degree of –NH_2_ groups on the chiro-optical parameters of the polymeric system was noted (Table 2). However, the change in the values of specific ellipticity ([æ]) is antipathic to the change in [α], e.g., despite the manifestation of the hypochromic effect of [æ] for both salt forms of CS relative to the individual antipodes of AspA, the absolute values of [æ] of CS·L-AspA and CS·D-AspA increase and decrease with increasing [AspA]:[–NH_2_], respectively. In quantitative terms, the observed effects are most pronounced for CS·L-AspA. A bathochromic shift in the wavelength of the dichroic band maximum (λ_0_) was also noted, both with an increase in the amount of acid unbound to the polymer and in comparison with L-(D-)AspA.

To exclude the influence of acid not associated with the polymer, AspA/CS systems with a molar ratio [AspA]/[–NH_2_] = 0.85 mol·(mol of NH_2_)^−1^ were used for further studies.

### 2.2. Optical Properties

To establish the spectral properties of our glycerohydrogel sol–gel plates and their components, studies of the optical transmission of these objects were carried out in combination with an assessment of the absolute refractive index and the average cosine of the scattering angle.

**Figure 3:**
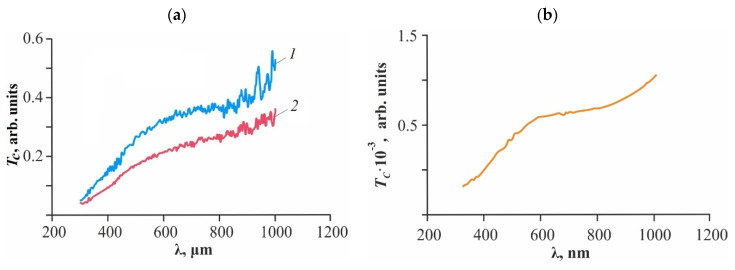

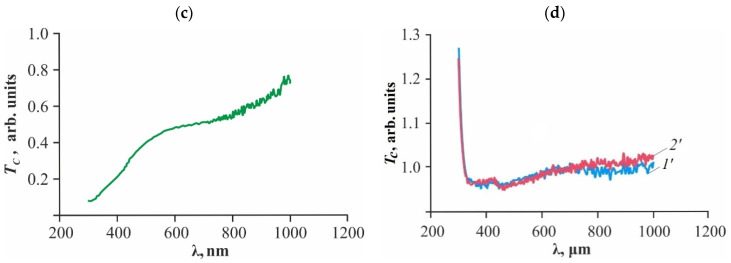
Collimated transmission spectra of our thin-film glycerohydrogel sol-gel plates based on CS·L-AspA (**a**, ***1***) and CS·D-AspA (**a**, ***2***), spectra of thin films of Si polyolate (**b**), glucomannan (**c**), and CS′·L-AspA (**d**, ***1′***) and CS′·D-AspA (**d**, ***2′***).

Collimated transmission spectra in the near-UV, visible, and near-IR spectral ranges show that our thin-film glycerohydrogel CS·L-(D-)AspA sol-gel plates absorb probing radiation over the entire range of wavelengths studied (Figure 3a). A comparative analysis of the spectra of the polymer substances that make up the plates indicates two key aspects. First, Si polyolate is the main absorbing component, which significantly affects the total values of collimated transmission of the glycerohydrogel material (Figure 3b). Glucomannan forms the general trend of the spectral line and absorbs intensely in the spectral range of 300–600 nm (Figure 3c). It should be noted that, according to the results of our $\langle \cos\theta \rangle$ measurements, these polymers are also the main scattering components of the glycerohydrogel system (Table 3). Second, individual salt complexes CS′·L-AspA and CS′·D-AspA demonstrate high transmittance of incident light (close to unity) in the studied range of λ, as well as the effect of optical clearing of the glass substrate within λ = 300–320 nm (Figure 3d). Such a high radiation permeability in the optical range and, in particular, in the near-UV range, in combination with the relative refractive index being close to unity (calculated from experimental values of the absolute refractive index, Table 3) indicates our successful choice of glass as a support material.

It is also worth noting the influence of the AspA isomer in the composition of salt complexes on the transmission spectrum of our glycerohydrogel sol-gel plates and thin films with their geometric identity. A significant discrepancy in the spectral values of *T*_c_ was observed within λ = 400–1000 nm and amounts to ~0.3 arb. units for CS·L-AspA and ~0.2 arb. units for CS·D-AspA, respectively (Figure 3a). For CS′·L-AspA and CS′·D-AspA, discrepancies in the *T_c_* = *f*(λ) spectra within this spectral range were also observed, but they were significantly smaller in comparison with CS·L-(D-)AspA (Figure 3d). The revealed differences cannot be caused by random fluctuations in the volume fractions of components in the irradiated volumes, which is confirmed by samples of objects of various thicknesses, averaging of spectral lines over several scans, and parallel experiments. It seems that the discrepancies in collimated transmission may be caused by differences in the supramolecular ordering of the polymeric substance of these samples, which predetermined the studies described below.

### 2.3. Surface Morphology

**Figure 4:**
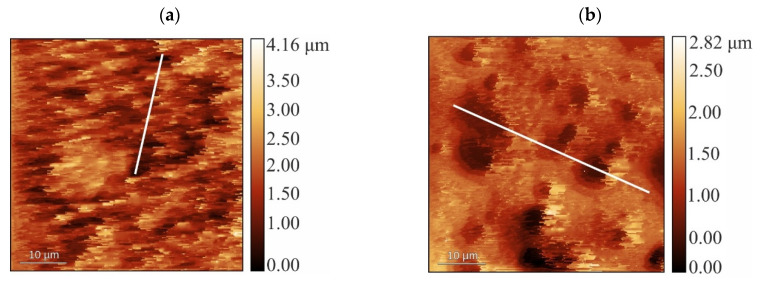

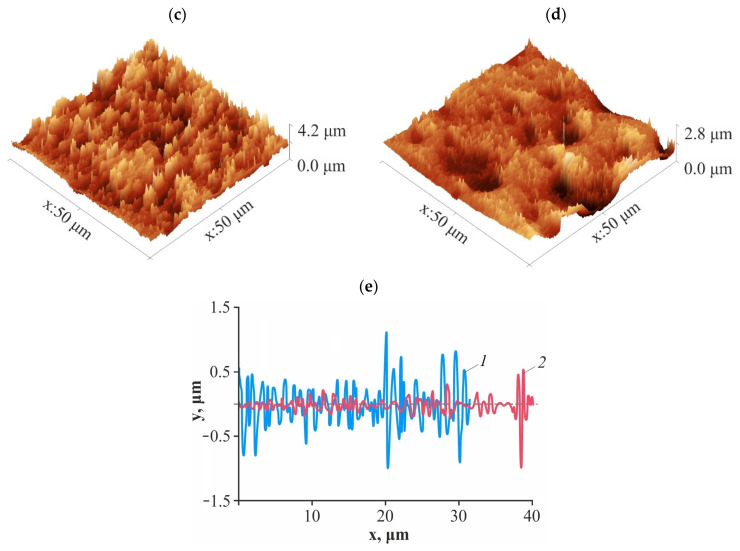
AFM images of the surface (**a**–**d**) and surface roughness profile ((**e**); the cross section is marked in (**a**,**b**) with the white line) of our thin-film glycerohydrogel sol-gel plates based on CS·L-AspA (**a**,**c**,**e**, ***1***) and CS·D-AspA (**b**,**d**,**e**, ***2***).

Atomic force microscopy (AFM) in a semi-contact mode made it possible to minimize mechanical damage to the soft surface of our CS·L-(D-)AspA glycerohydrogel sol-gel plates and to analyze their microrelief. Both samples of enantiomeric CS salt complexes in the glycerohydrogel composition are characterized by a complex surface topography (Figure 4a–d). Two types of structural irregularities are observed, namely needle-like supramolecular formations (protrusions) and granular pores of a spherical or close to spherical shape (depressions). For CS·L-AspA-based plates, a predominantly needle-like relief is visualized, with a predominance of protrusions up to 4.2 µm high and a horizontal pitch of ~2–7 µm, whilst for CS·D-AspA-based plates, a needle-grained relief is visualized with protrusions up to 2.8 µm high, horizontal step of ~1–5 µm, and depressions (pores) with diameters of ~3–10 µm.

Analysis of cross sections of these AFM scans revealed differences in the values of average roughness and root-mean-square surface roughness, as well as in the values of asymmetry and kurtosis of the samples depending on the AspA enantiomer (Figure 4e, Table 4). The sample of our hydrogel sol-gel plates based on CS·L-AspA has the most developed surface with the greatest height of roughness, height of protrusions, and depth of depressions. The surface of the CS·D-AspA sample is significantly more asymmetrical than that of the CS·L-AspA sample and has a larger profile distribution (kurtosis).

### 2.4. Supramolecular Ordering

X-ray diffractometry at large scattering angles was applied to CS·L-(D-)AspA powders lyophilically isolated from the corresponding sol–gel plates without preliminary removal of the structure formers (glucomannan and Si polyolate) and the plasticizing agent (glycerol). Significant differences in the supramolecular ordering were revealed in comparison with the original CS sample and CS′·L-(D-)AspA powders lyophilically isolated from the corresponding solutions. They also depend on the isomeric form of the chiral acid residue.

**Figure 5:**
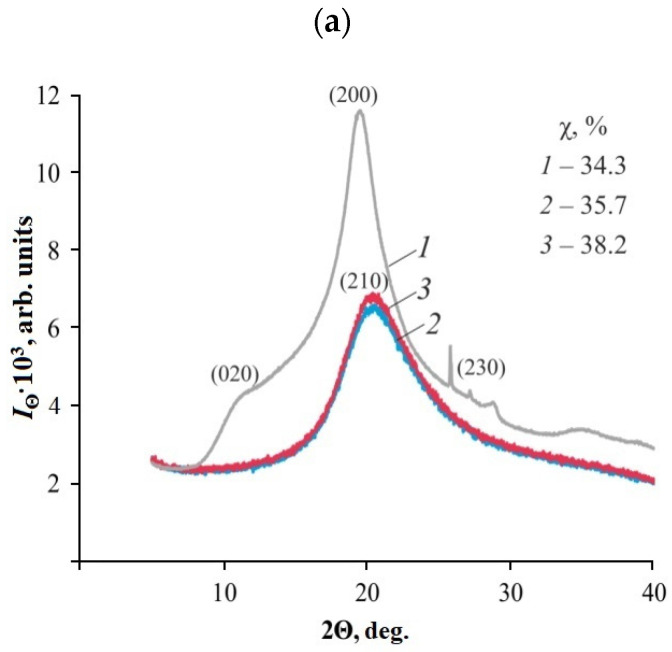

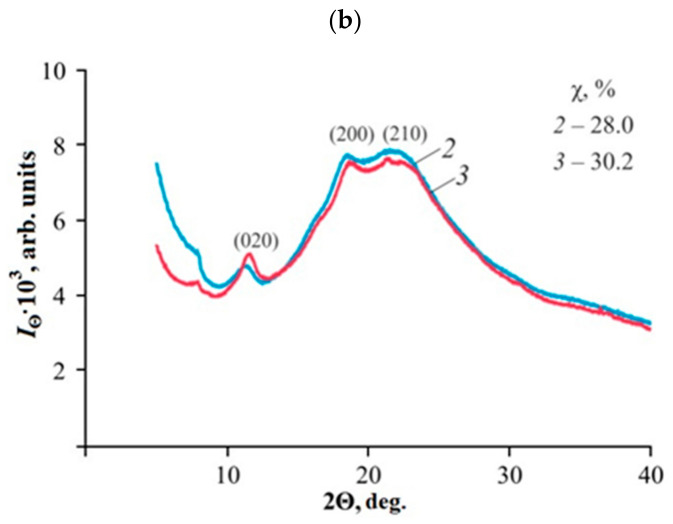
X-ray diffraction patterns of the powders: initial sample of CS (**a**, ***1***); CS·L-AspA (**a**, ***2***) and CS·D-AspA (**a**, ***3***) isolated from our thin-film glycerohydrogel sol-gel plates; CS′·L-AspA (**b**, ***2***) and CS′·D-AspA (**b**, ***3***) obtained from solutions.

The diffraction patterns of CS·L-(D-)AspA powders are typical for amorphous–crystalline polymers with a low crystallinity degree [42,43]. It is noteworthy that the equatorial X-ray profiles of enantiomeric CS·L-(D-)AspA salts lose the (020) and (230) reflections detected in the diffraction patterns of the original CS powder (Figure 5a). In addition, instead of the reflection (200) with a maximum at 19.4 deg, a less intense reflection (210) appears at 21.8 deg. This indicates, firstly, that the supramolecular structure of the powders isolated from the plates is represented by almost a single polymorphic modification. Second, smaller (compared to CS) structural elements are formed in CS·L-(D-)AspA, in whose crystal structure there are almost no water molecules. The X-ray diffraction patterns of CS′·L-(D-)AspA powders reveal at least three polymorphic modifications of the polymer (020, 200, and 210) (Figure 5b). Some blurring of the equatorial reflections (200) and (210) may indicate that the orderliness of the arrangement of macromolecular sections in the direction perpendicular to the orientation axis is limited to a small number of neighbors. Nevertheless, the reflection (210, ~22.2 deg) indicates the presence of a dehydrated polymorph in the structure of these samples. Thus, in both salt samples, there are polymorphs of the hydrated and anhydrous forms of the polymer, which, according to the classification of K. Ogawa et al. [44,45], allows us to classify CS·L-(D-)AspA and CS′·L-(D-)AspA as type I non-hydrated salts, where water molecules are replaced by acid anions.

Protonation of glucosamine CS units is known to eliminate the possibility of amino groups participating in hydrogen bonding and, due to intramolecular electrostatic repulsion, partially weakens the initial rigid crystalline structure of the polymer. However, CS·L-(D-)AspA and CS′·L-(D-)AspA show a compacted supramolecular orientation of macrochains, as evidenced by the high values of the crystallinity degree (χ) of the samples (Figure 5), which are not typical for CS salt complexes with traditional carboxylic acids (CH_3_COOH, HCOOH), whose χ value, as a rule, does not exceed 25% [38,42,43]. The χ values of the samples isolated from our glycerohydrogel plates exceed those of the original CS powder. It seems that the formation of a relatively highly ordered structure of CS·L-(D-)AspA and CS′·L-(D-)AspA may be due to the previously discovered effect of counterionic condensation in CS solutions in AspA [39]. In this process, polycation association with counterions of the acid residue prevails over the dissociation of ionogenic groups, which is accompanied by phase segregation of the polymeric substance at the level of nanoparticles. Most likely, the most favorable conditions for the occurrence of associative processes are realized in the environment of a hybrid organic–inorganic network rather than in an aqueous medium (Figure 1). The detected structural changes are most pronounced for the D-enantiomeric salt complexes of CS, and do not depend on the initial system which the samples were isolated from. The highest crystallinity values were also observed for CS D-aspartate.

**Figure 6:**
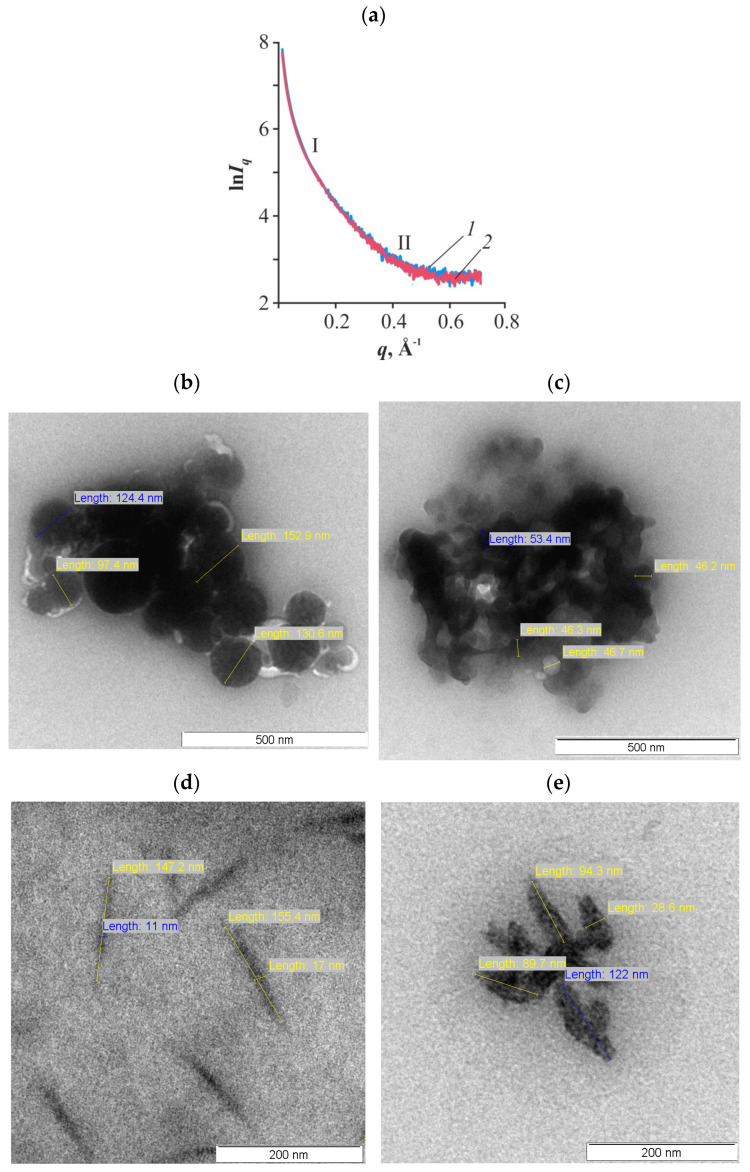
Small-angle X-ray scattering curves (scattering indicatrices) of our thin-film glycerohydrogel sol-gel plates based on CS·L-AspA (**a**, ***1***) and CS·D-AspA (**a**, ***2***) in Guinier’s coordinates. TEM images of nanoparticles and nanorods of CS·L-AspA (**b**,**d**) and CS·D-AspA (**c**,**e**).

Small-angle X-ray scattering was used for structural diagnostics of the supramolecular ordering of the polymeric substance in our thin glycerohydrogel CS·L-(D-)AspA sol-gel plates in the nanometer range. The SAXS intensity curves in the semilogarithmic coordinates of both samples of chiral salts are symbathic, have the form of smoothly decreasing dependences with the modulus of the scattering wave vector, and have no Bragg peaks (Figure 6a). This indicates the scattering of loosely packed polydisperse domains of inhomogeneities and the amorphous structure of the samples, which is consistent with X-ray diffraction data at large angles (Figure 5a). The scattering indicatrix ln *I_q_* = *f*(*q*) reveals two straight sections (I and II), differing in the slope of the radiation intensity *I_q_* ~ *q*^−*n*^. The first one, at which the fastest decrease in intensity *I*(*q*) is observed, corresponds to the range of scattering coordinates *q* < 0.15 Å^−2^. The second corresponds to the range of the modulus of the scattering vector *q* ~ 0.25–0.45 Å^−2^. The crossover point can be considered *q* ~ 0.2 Å^−2^. The power-law decay index at small *q* values for the CS·L-AspA and CS·D-AspA samples is *n* = 1.3 and 1.2, respectively, which indicates the presence of scattering bulky domains in the form of hollow spheres in the spatial network of the hydrogel (Table 4). In the range of higher *q*, the slope of the SAXS curve decreases down to *n* = 0.5 and 0.3, which corresponds to the rod-shaped shape of the scattering inhomogeneities. Differences are also noted in the quantitative indicators of the average radius of gyration of the disclosed phase inhomogeneities in the supramolecular structure of our hydrogel sol-gel plates, e.g., the CS·D-AspA sample is characterized by a smaller average size of scattering aggregates and a narrower range of variations in their values (Table 4). A similar effect of the isomeric form of the acid residue on the fractal dimension of scattering objects in the nanometer range and the average characteristics of scattering domains was discovered for glycerohydrogel plates of CS·L-(D-)ascorbate [47].

In general, our analysis of SAXS indicatrices shows that the supramolecular structure of the soft condensed phase of the polymeric substance in the glycerohydrogel composition is represented by two types of scattering inhomogeneities, namely hollow spheres and rod-shaped particles. The shape of the scattering domains predicted by SAXS was visualized using transmission electron misroscopy (TEM), e.g., spherical CS·L-AspA structures are polydisperse nanospheres with a size distribution within 60–120 nm and an average diameter of about 90 nm, while CS·D-AspA structures are relatively monodisperse nanoparticles with sizes in the range of 45–55 nm and an average diameter of 50 nm (Figure 6b,c). The rod-shaped structures of CS·L-AspA are represented by anisometric particles with lengths of ~100–160 nm and widths of ~10–20 nm, with values for CS·D-AspA being ~85–125 nm and ~20–30 nm (Figure 6d,e).

Previously, we discovered, using SEM, that spherical and ellipsoidal nanoparticles are formed in the CS + L-AspA + H_2_O system due to ion–ion associations, whose elemental composition is represented by the salt form of the polymer (CS·L-aspartate) [39,40]. Taking into account the results of these works and the present SAXS and TEM studies, it can be stated that, in contrast to the optical properties in the UV, visible, and near-IR ranges (Section 2.2), CS·L-(D-)AspA is the main scattering component of our glycerohydrogel plates in the X-ray range.

## 3. Conclusions

For the first time, the optical properties of chiral nanostructured sol–gel plates of chitosan L- and D-aspartate were studied in a wide range of wavelengths, and the structural features of the glycerohydrogel material were characterized. The totality of the results obtained allows us to state that the chiro-optical characteristics, collimated transmission, average cosine of the scattering angle, roughness profile, and surface asymmetry, as well as the degree of crystallinity of the polymeric substance and the morpho-dimensional organization of phase inhomogeneities in our glycerohydrogel sol-gel plates and the individual components included therein, are determined not only by the spectral range of electromagnetic waves but also by the enantiomeric form of the acid residue.

For example, the most significant contribution to the total optical activity of the objects under study is made by the induced asymmetric chromophores (${\text{–}\mathrm{NH}}_{3}^{+}$·HAsp^−^) in the polymer chain. The ensemble of excited CS·L-AspA or CS·D-AspA molecules exhibits a positive or negative dichroic effect of circular polarization, respectively. CS·L-AspA shows a higher absorption capacity of the collimated radiation flux, while CS·D-AspA is characterized by higher scattering power. CS·L-AspA sol-gel plates have a more developed surface relief, whilst CS·D-AspA plates have a more asymmetrical distribution of surface irregularities. Both CS·L-(D-)AspA samples are characterized by a more compacted supramolecular structure, but the highest values of the degree of crystallinity were observed for CS D-aspartate. Polydisperse and larger nanospheres and anisometric particles were found in our CS·L-AspA sol-gel plates, which is confirmed by the direct TEM visualization of nano-sized structures.

The proximity to 1 of the transmittance of probing radiation of CS L- and D-aspartates deposited on glass substrates and the effect of optical clearing of these samples in the near-UV range open up new possibilities for the applications of chitosan-containing materials in sensorics and photonics. In particular, our CS·L-(D-)AspA sol-gel plates can be used in medico-biological applications such as optical bioimaging, both in situ and in vivo, of cellular and body bio-objects and dynamic biological processes, as well as optical diagnosis and therapy, selective UV sterilization, etc.

## 4. Materials and Methods

### 4.1. Materials

The starting reagents were CS with a viscosity-average molecular mass ${\bar{M}}_{\eta }$ = 200 kDa, a degree of deacetylation 82 mol%, and a moisture content of *W* = 8 ± 1 wt% (Bioprogress Ltd., Shchyolkovo City, Russia); glucomannan with ${\bar{M}}_{\eta }$ = 1100 kDa (Uspekh LLC, Moscow, Russia); L-AspA with 99% basic substance (JSC Bioamid, Barnaul, Russia); D-AspA with 98% basic substance (Vekton Corp., Moscow, Russia); Si(OGly)_4_ ∙ 3 GlyOH (58.7 wt%, Ural Branch of Russian Academy of Sciences, Institute of Organic Synthesis named after I.Ya. Postovsky, Yekaterinburg, Russia); distilled water (Milli-Q, pH = 6.0); and ethyl alcohol (C_2_H_5_OH, 95.6%, Baza Khimreaktivov Corp., Moscow, Russia). All reagents were chemical grade and used without further purification.

### 4.2. Preparation of the Objects of Study

#### 4.2.1. Preparation of Solutions

To obtain solutions of chitosan L-(D-)aspartate (CS·L-(D-)AspA), calculated weighed portions of CS (considering its *W*) and L-(D-)AspA were placed into a volume of H_2_O and stirred on a magnetic stirrer at a rotation speed of 400–500 rpm at 50 °C until visual dissolution of the powders (2–3 h). The concentrations of the polymer and acid in the solution varied in the ranges of *C*_CS_ = 0.05–0.6 g·dL^−1^ and *C*_AspA_ = 0.1–0.8 g·dL^−1^, respectively. The quantitative composition of salt CS·L-(D-)AspA complexes was expressed as the molar ratio [AspA]:[~–NH_2_] (mol·(mol of NH_2_)^−1^). Before experiments, all solutions were filtered through a Millipore filter with a pore diameter ≤ 0.45 μm and kept for 24 h to remove air bubbles.

To prepare a glucomannan solution with a concentration of 0.2 g·dL^−1^, the calculated amount of H_2_O was added to an alcohol suspension of the polymer (volume ratio glucomannan:C_2_H_5_OH = 1.0:0.5) and stirred on a magnetic stirrer at a rotation speed of 500–600 rpm at 25 °C for 10 min, followed by heating to ~80 °C using microwave radiation with a power of 800 W (4 times for 30 s) to remove ethyl alcohol.

#### 4.2.2. Preparation of Glycerohydrogel Plates, Thin Films, and Powders

To obtain glycerohydrogel plates, aqueous solutions of CS·L-AspA or CS·D-AspA with *C*_CS_ = 0.6 g·dL^−1^ and *C*_AspA_ = 0.4 g·dL^−1^, an aqueous glucomannan solution, and a glycerol Si(OGly)_4_ solution were mixed in a mass ratio of components 1:1:0.5 and thoroughly homogenized with a glass rod for 1–2 min (Figure 1a). The finished mixture composition was transferred onto a horizontal glass substrate (preliminarily defatted with C_2_H_5_OH) at a rate of 0.4 mL·cm^–2^ and kept at room atmosphere for ~72 h until complete gelation. The thickness of the CS·L-(D-)AspA glycerohydrogel sheets was *d* = 500 ± 30 µm.

The procedure for obtaining thin films from CS L-(D-)aspartate, glucomannan, and Si polyolate, and the concentrations of the solutions of CS·L-(D-)AspA, glucomannan, and Si(OGly)_4_ ∙ 3 GlyOH, were similar to those for glycerohydrogel plates. In the case of the formation of films from aqueous solutions of polymers, the completeness of water removal and the formation of an air-dry film sample were controlled gravimetrically. Samples of the salt complexes isolated from initial solutions, i.e., in the absence of glucomannan and Si polyolate, will be further denoted as CS′·L-(D-)AspA.

Powders of the CS·L-(D-)AspA salts were obtained from glycerohydrogel plates, and CS′·L-(D-)AspA powders were prepared from the corresponding solutions of the same concentration as when obtaining plates by lyophilization on a BenchTop 2 K (VirTis, Los Angeles, CA, USA) at −57 °C and pressures of 2–5 mmHg.

### 4.3. Methods

#### 4.3.1. Potentiometric Titration

The protonation degree ($\alpha^{\prime }$, mol.%) of CS in aqueous solutions of L-(D-)AspA was determined by the potentiometry method on an automatic Mettler Tolledo G20 titrator (MTD, Saarbrückent, Germany) with a glass electrode at 23 ± 1 °C. 0.25 g·dL^−1^. CS solutions in L-(D-)AspA and a 0.05 N NaOH solution were used, with an accuracy of 0.1 mol.%, for three replicates. The dissociation degree ($\alpha^{\prime \prime }$) of L-(D-)AspA in an aqueous solution was determined conductometrically in conditions of equilibrium H_2_Asp ↔ H^+^ + HAsp^−^, taking into account the basicity of the acid [41]. The values of α′ were calculated by the formula:

(1) $$\alpha^{\prime }=\frac{\left[–NH_{3}^{+}\right]}{\left[–NH_{3}^{+}\right]+\left[–NH_{2}\right]}\cdot \alpha^{\prime \prime }\cdot 100=\frac{\alpha^{\prime \prime }\cdot 100}{1+\left(\frac{\left[H^{+}\right]\left[–NH_{2}\right]}{\left[–NH_{3}^{+}\right]}\right)/\left[H^{+}\right]}=\frac{\alpha^{\prime \prime }\cdot 100}{1+10^{\mathrm{pH}\;-\;p{Ka}_{2}}}.$$

Hydrogen indicator (pH) was measured on a Mettler Toledo Five Easy Fe20 pH meter (MTD, Singapore), with an accuracy of ±0.01 pH and five replicates.

Gravimetric measurements were carried out on Ohaus Adventurer AR 1530 scales (with a weighing accuracy of ±0.00002 g).

#### 4.3.2. Spectroscopic Methods

ORD spectra were recorded on an SPU-E spectropolarimeter (RF) with a high-pressure DRSh-250 lamp (RF) in a heat-fixed cuvette with quartz windows (RF) within λ = 280–710 nm at 25 °C. The error in measuring rotation angles did not exceed ±0.002°, and three replicates were taken. ORD spectra were represented in units of specific optical rotation [α].

CD spectra were recorded on a Chirascan™ spectrometer (Applied Photophysics Ltd., Charlotte, NC, USA) with a UV detector and a 150 W xenon lamp in a thermostated quartz cuvette “Hellma Analytics” (Mülheim, Germany) within λ = 200–600 nm at 25 °C. The scanning mode was 1 nm, the detection time was 0.5 s, and two replicates were taken. CD spectra were processed using Pro-Data Suite control software and expressed in units of specific ellipticity [æ].

Specific optical rotation [α] (deg·mL·dm^−1^·g^−1^) and specific ellipticity [æ] (deg·mL·dm^−1^·g^−1^) were calculated from Equations (1) and (2), respectively:

(2) $${\left[\alpha \right]}_{\lambda ,\;\mathrm{nm}}^{25{}^{\circ}C}=\frac{(\alpha -\alpha )\cdot 100}{C_{\mathrm{CS}}\cdot l}$$

(3) $${\left[\text{æ}\right]}_{\lambda ,\;\mathrm{nm}}^{25{}^{\circ}C}=\frac{(æ-{æ}_{0})\cdot 100}{C_{\mathrm{CS}}\cdot l},$$

where α (æ) and α_0_ (æ_0_) are the measured angles of optical rotation (light ellipticity) of the solution and solvent (H_2_O), respectively, deg; *C*_CS_ is the concentration of the solution, g·(100 mL)^−1^; *l* is the optical path length, 1 dm. The concentration of the working solutions for ORD and CD was *C*_CS_ = 0.5 g·dL^−1^ and *C*_CS_ = 0.6 g·dL^−1^, respectively.

#### 4.3.3. Optical Methods

Optical transmission spectra were recorded on a collimating system which included an Ocean Optics QE65000 spectrometer (Ocean Insight, Largo, FL, USA), two Ocean Optics 74DA collimators, and an Ocean Optics DH-2000-BAL halogen–deuterium lamp at 23 ± 1 °C. To connect the collimator with a broadband radiation source and a spectrometer, an Ocean Optics P100-2-UV-VIS fiber optic patch cord and a P100-1-UV-VIS patch cord were used, respectively. The exposure time was 50 ms and averaging was carried out over 5 consecutive scans. Emission spectra were recorded at 5 points on the sample surface, followed by averaging. The collimated transmission coefficient (*T*_c_) was estimated as the ratio of the average transmission spectrum of the sample to the transmission spectrum of the holder (glass). The measurement error did not exceed 5%.

To determine the average cosine of the scattering angle ($\langle \cos\;\theta \rangle$), a GN-5P He–Ne laser with a radiation wavelength of 632 nm (LLC Plasma, Skopje, Russia) was used; the beam diameter being 1 mm. The technique consisted of obtaining a scattering pattern when monochromatic radiation passed through the sample deviated from the normal, which was digitized to obtain actual dimensions. The digitized scattering pattern demonstrated the isotropic behavior of the medium; the radius of the scattering spot was set along the boundary of the scattered light intensity distribution, corresponding to half the maximum. The approach used differs from the classical method for $\langle \cos\theta \rangle$ calculation when deriving the diffusion equation in the theory of diffusion approximation [49]; however, it is very informative for a comparative analysis of the main scattering components of the samples under study.

The refractive index was measured on an IRF-454 B2M refractometer at λ = 584 nm and 23 ± 1 °C.

#### 4.3.4. Microscopy

AFM images were obtained on a scanning probe NTEGRA Spectra microscope (NT-MDT Ltd., Zelenograd City, Russia) in quasi-hopping mode. An FMG01 cantilever (Tipsnano, Tallinn, Estonia) with a rigidity of 2.5–10 N·m^−1^, a resonant frequency of 115–190 kHz, and a tip radius of 10 nm was used. Samples were attached to a magnetic holder using double-sided tape. Construction of the roughness profile and extraction of roughness parameters of the samples were carried out using Gwyddion software, v. 2.56. The number of processed sections of AFM profiles was at least 10 for each sample.

TEM images were obtained on a Libra 120 Karl Zeiss transmission electron microscope (Oberkochen, Germany). The accelerating voltage was 120 kV and the magnification was (4–20)·10^3^. Solutions with *C*_CS_ = 0.05 g·dL^−1^ and *C*_AspA_ = 0.1 g·dL^−1^ were used, which were applied to copper grids with a formvar substrate.

#### 4.3.5. X-ray Powder Diffraction

X-ray diffraction patterns were obtained on a DRON-8T diffractometer (JSC IC Burevestnik, Saint-Petersburg, Russia) with CuK_α_ radiation, a parabolic Göbel mirror (AXO Dresden GmbH, Dresden, Germany), and a position-sensitive detector Mythen 2R1D with 640 channels (Dectris, Baden-Daettwil, Switzerland). Resolution was 2Θ = 0.0144 deg in a 2 mm quartz cuvette. Focus beam geometry: axial slits of 12 mm and equatorial slits of 0.25 mm, respectively. Registration was carried out in the angle range 2Θ = 5–40 deg at points with a step of 0.02 deg for the central channel of the detector and an exposure time of 10 s at each point. The degree of crystallinity (χ, %) was calculated as the ratio of the integral intensity of the total scattering of crystallites to the total scattering from amorphous and crystalline regions by graphical integration (QCAD 3.15 program) [28].

#### 4.3.6. Small-Angle X-ray Scattering

Small-angle X-ray scattering (SAXS) measurements were carried out on a D8 Discover small-angle diffractometer (Bruker, Billerica, MA, USA) with CuK_α_ radiation and a LynxEye solid-state position-sensitive detector in 0D mode. The slit width in front of the detector was 0.2 mm. The scattering intensity *I*_q_ was recorded in the range of the modulus of the scattering wave vector *q* = 0.2–7.1 Å^−2^, where *q* = 4π sinΘ·λ^−1^, 2Θ being the scattering angle (deg) and λ the radiation wavelength (1.542 Å). The structural parameter *n*, which characterizes the morphology of scattering inhomogeneities, was determined from the tangent of the slope of the rectilinear sections of the scattering curve (ln *I_q_*; *q*). The average radius of gyration *R_n_* of scattering inhomogeneities (in our case, having the physical meaning of the average linear size of scattering domains) was from the dependence in Guinier coordinates (ln *I_q_*; *q*^2^) and calculations using Equation (3) [50]:

(4) $$I_{q}\left(q\right)=I\left(0\right)\exp\left(\frac{-q^{2}R_{n}^{2}}{3}\right).$$

The assignment of *n* to the shape of scattering inhomogeneities was carried out according to the method reported elsewhere [51].

#### 4.3.7. Statistical Analysis

Statistical analysis was performed for three (as a minimum) different replicates of each experiment. The results are presented as mean values ± standard deviation (*n* ≥ 3). The significant difference was evaluated by an unpaired two-sample Student’s *t*-test. The difference was considered statistically significant when *p* < 0.05.

## Figures and Tables

**Table 1:** Possible uses of chitosan-based thin-film nanostructured composites.

No.	Application	References
1.	Flexible conductive film substrates for innovative energy accumulators and sources	[1,2]
2.	Wearable electronic devices	[3,4]
3.	Self-healing electrical sensors	[5]
4.	Highly sensitive optical sensors to diagnose biological macromolecules (DNA, proteins), cells, and genetic markers	[6,7]
5.	Sensors for optical visualization of biological objects	[8]
6.	Sensors for drug detection	[9]
7.	Sensors for detection of other small organic molecules	[10,11]
8.	Sensors for detection of trace amounts of heavy metal ions	[12,13]
9.	Luminescent and colorimetric sensors for ecological monitoring of the environment and food	[14]
10.	Active hydrogel layers of mesoporous sol–gel adsorbents	[15]
11.	Highly effective integrated optical humidity sensors	[16,17]
12.	UV detectors operating at high frequencies	[18]

**Table 2:** Physicochemical and chiro-optical parameters of aqueous solutions of L-(D-)AspA and CS∙L-(D-)AspA at 25 °C.

CS Concentration *C*_CS_, g·dL^−1^	AspA Concentration *C*_AspA_, g·dL^−1^	Molar ratio [AspA]:[–NH_2_], mol·(mol of NH_2_)^−1^	Parameters			
			CS∙L-AspA	CS∙D-AspA	CS∙L-AspA	CS∙D-AspA
Acid–base indicators			pH		Protonation degree α′, mol%	
0.25	0.2	1.0	4.3	4.3	26.8	32.8
	0.4	2.0	3.5	3.5	16.8	23.7
	0.8	4.0	3.1	3.0	7.5	9.2
Spectral CD characteristics			Wavelength of the dichroic band maximum λ_0_, nm		Specific ellipticity at λ_0_ [æ]_max_·10^−5^, deg·mL·dm^−1^·g^−1^	
–	0.5	–	205	206	17.8	−16.5
0.6	0.4	0.85	214	214	9.6	−11.7
	0.6	1.0	215	218	10.8	−11.8
	0.8	2.0	219	220	10.6	−10.9

**Table 3:** Refractive index and geometric-optical parameters of our glycerohydrogel CS·L-AspA sol-gel plates and of thin films of the individual components of the plates. 23 ± 1 °C.

Sample	Refractive Index	Thickness *d*, μm	Scattering Angle	
			$\langle \cos\theta \rangle$	θ, deg
Plates				
CS·L-AspA + glucomannan + Si polyolate	1.5101	500 ± 30	0.9961	5.0619
CS·D-AspA + glucomannan + Si polyolate	1.5110	500 ± 30	0.9926	6.9746
Films of the plates’ individual components				
CS′·L-AspA	1.5208 *	50 ± 5	0.9992	2.2919
CS′·D-AspA	1.5189 *	50 ± 5	0.9994	1.9849
Glucomannan	1.5170	250 ± 30	0.9951	5.6743
Si polyolate	1.5438	1050 ± 30	0.9973	4.2113
Support				
Glass	1.5141	1050 ± 30	0.9984	3.2416

* The refractive index values are consistent with those reported by Azofeifa et al. [24].

**Table 4:** Structural and dimensional characteristics of the surface and supramolecular ordering of the polymer substance of our glycerohydrogel CS·L-AspA and CS·D-AspA sol-gel plates according to AFM and SAXS data.

Parameter		Sample	
		CS·L-AspA	CS·D-AspA
AFM			
Average roughness *R_a_*, µm		0.13 ± 0.09	0.06 ± 0.01
Root-mean-square roughness *R_q_*, µm		0.18 ± 0.11	0.09 ± 0.02
Asymmetry *R_sk_*		−0.02 ± 0.39	0.09 ± 0.7
Kurtosis *R_ku_*		6 ± 1	13 ± 8
Maximum roughness height *R_t_*, µm		1.4 ± 0.7	1.0 ± 0.6
Maximum protrusion height *R_p_*, µm		1.1 ± 0.4	0.5 ± 0.3
Maximum depression depth *R_v_*, µm		1.0 ± 0.3	0.9 ± 0.2
SAXS			
Power-law decay index of ln *I*(*q*) = *f*(*q*), *n*	I	1.3	1.2
		hollow spheres	
	II	0.5	0.3
		rods	
Average radius of gyration of scattering inhomogeneities, *R_n_* (Å)		10–50	15–30

## Data Availability

The raw/processed data can be provided when required.
